# The Methacrylate Adhesive to Double-Lap Shear Joints Made of High-Strength Steel—Experimental Study

**DOI:** 10.3390/ma12010120

**Published:** 2019-01-01

**Authors:** Marta Kałuża, Jacek Hulimka, Jan Kubica, Marcin Tekieli

**Affiliations:** 1 Department of Structural Engineering, Faculty of Civil Engineering, Silesian University of Technology, Akademicka 5 St., 44-100 Gliwice, Poland; jacek.hulimka@polsl.pl (J.H.); jan.kubica@polsl.pl (J.K.); 2 Institute for Computational Civil Engineering, Department of Civil Engineering, Cracow University of Technology, Warszawska 24 St., 31-155 Kraków, Poland; mtekieli@l5.pk.edu.pl

**Keywords:** methacrylate adhesive, steel-to-steel joint, flexible adhesive, high-strength steel, double-lap joint

## Abstract

In typical technical applications, steel components are usually connected by welding or with mechanical connectors. An alternative solution, typical in the aviation and automotive industry, but not widespread in engineering structures, is to join thin sheet metal using adhesives. The article presents an experimental study of adhesive joints used in overlap connections subjected to static tension. A methacrylate adhesive, selected experimentally from a range of adhesives, which combines the optimum strength and strain properties, was tested. The laboratory tests were carried out on double-lap specimens made of high-strength Domex 700 steel. On the basis of the experimental results, the behavior of the specimens and their failure mechanism, depending on the anchorage lengths used (200, 300 and 400 mm), are described. The tests confirmed the effectiveness of the selected methacrylate adhesive in a practical application. It was shown that with the appropriate anchorage length (adequate to the type of steel components and the joint geometry) between 300 and 400 mm, the capacity of the adhesive joint is higher than the capacity of a single steel component. Two types of specimen behavior were recognized: Quasi-brittle, which occurs at the anchorage length of 200 mm, and ductile, observed for 300 mm and 400 mm anchoring. In addition, thanks to the optical measurement method used, a detailed strain distribution on the specimen surface was determined. The data will be used for subsequent validation of an analytical and numerical model.

## 1. Introduction

For many years, steel has been the basic construction material used in almost all industrial sectors. Welded, bolted (also using preloaded bolts), or riveted connections have been very popular and often used in civil engineering structures. Their effectiveness is confirmed by a number of studies and analyses, including numerical simulations [1,2,3]. Unfortunately, each of these joint types introduces notches in the form of zones with different mechanical properties (welded joints) or openings interrupting material continuity (bolts). For structures subjected to static loads, such notches are relatively easy to consider in the assessment of load-bearing capacity and changes in stiffness. A much worse situation is in the case of components and structures subjected to dynamic or cyclic loads [4,5].

To reduce or completely eliminate problems related to the use of mechanical connections, adhesive joints constitute a good alternative [6]. The idea of using adhesives for steel joints is not new. There have been many theoretical analyses, which describe the mechanism of coupling and failure modes of adhesively joined elements [7,8,9]. The extensive experience in gluing structures used in aviation and automotive industries are well known, but in the case of engineering structures (e.g., machine or construction industry), so far, the attempts to use adhesives are extremely rare. The mechanical joints (especially welded joints) are still prevailing, while adhesive joints have become popular, mainly in the case of the strengthening of steel structures.

Typical strengthening of steel components, in civil engineering structures where static or quasi-static loads dominate, is made using FRP (fibre reinforced polymer) materials, which are used as an additional reinforcement applied on the epoxy resin adhesives dedicated to specific manufacturer-specific systems [10,11]. These adhesives feature high tensile and shear strengths, but show very low flexibility. Their application together with FRP strengthening on stiff elements (reinforced concrete beams, stiff steel beams, or columns) is justified and effective [12], because joined components have low elongation limits, and, thus, they tolerate the use of adhesives with low deformability. The same situation (with positive results) applies to the use of an epoxy to strengthen a thin metal profile on a relatively short length, where the deformability of the adhesive is of lesser importance [13].

The situation is completely different for steel-to-steel (or generally metal-to-metal) joints made by thin or flexible elements (thin steel beams, cold-formed profiles, and metal sheets), which are connected on a relatively long length or when a load acts not statically, but cyclically or dynamically. In such connections, the basic parameter is the deformability (or rigidity) of the adhesive [14]. It must be high enough to ensure a proper adhesive effort, level over the entire bonding length (involving the entire bond length to cooperate in transferring stresses [15]). This, by definition, eliminates stiff epoxy adhesives (typical for the construction industry), whose application leads to the stress concentration and brittle failure of the joint within a relatively short section [16]. At the same time, too high a deformability of the adhesive results in excessive mutual displacement (slip) between joined components. The ideal solution is a high-strength adhesive with simultaneous deformability, which guarantees durable and safe connection of steel components, while maintaining the required flexibility, without excessive movement [17]. Therefore, proper selection of an adhesive is a key issue, both with steel-to-steel [18,19] and steel-to-FRP connections [20]. Another solution, which allows the ensuring of an effective connection, is the usage of mixed joints that combine traditional methods and gluing [21,22].

Among adhesives that combine these two features (high-strength and appropriate deformability), a group of methacrylate adhesives (MA) was selected [20]. After performing the preliminary tests, one of them was finally selected as an appropriate one for further testing [19].

The selected adhesive was tested on typical overlap connections made of high-strength steel subjected to static (the presented paper) and cyclic (will be presented in a separated paper) loading. The main aim of the presented work is the experimental confirmation of the possibility of effective use of commercially available methacrylate adhesive in a typical engineering application, i.e., to form a structural connection of elements made of high-strength steel. Studies of three anchorage lengths facilitated the selection of the appropriate bonding length (for the high-strength steel and specimen dimensions used) that ensured the load-bearing capacity of the adhesive joint was higher than the capacity of a single steel component. Two failure mechanisms were distinguished related to the behavior of the specimens, which depends on the anchorage length. The additional aim of the experimental study was to obtain results for further validation of an analytical and numerical model. The necessary adhesive parameters required in these analyses were tested independently.

## 2. Methodology of Laboratory Tests

### 2.1. Test Specimens

Double-lap type specimens were used in the experimental investigation. Three types of specimens were subjected to static tension. All specimens consisted of two steel plates (made of raw steel considered as basic plates bonded adhesively by two raw steel plates, referred to as overlaps. The dimensions of the basic plates were 90 × 6 × 550 mm, while the cross-section of the overlaps was 50 × 6 mm with various lengths of 650, 550, and 450 mm, which resulted in three anchorage lengths of 400, 300, and 200 mm. The steel surface was prepared before glueing by mechanical pre-conditioning and degreased.

The overlaps were glued to the surface of the basic plates using a methacrylate adhesive (ITW Plexus Structural Adhesives, Rushden, UK). The selection of the glue was described in [19]. It was vitally important to ensure a constant thickness of the adhesive of 1.2 mm along the whole anchorage length. This was obtained using steel balls with a diameter of 1.2 mm, which were placed at several points along the length of the adhesive layer. 

The specimens marked as P1 (part one) were made using adhesive right after purchase. The glue used in specimens marked as P2 (part two), before application, was stored in the laboratory (in room temperature) for one year.

The test specimens were provided with the following note: S—static test, DL—double-lap specimen, MA—methacrylate adhesive, RS—raw steel, anchorage length—i—the order of the test in a given group, P1 (or P2)—part of the adhesive. Table 1 summarizes all tested specimens.

The general geometry of the specimens was similar to those given in the ASTM D3528-96 recommendation [23], but the overall size of the components was larger, so their proportions were different—which results from the requirements set by the cooperating research company (see: Acknowledgments). The reason for choosing such large elements was the recognition of the problem of adhesive lap joints in actual structures (with the dimensions close to the real steel element) and an analysis of an effective anchorage length in key elements, i.e., taken from the structural engineering.

**Figure 1:**
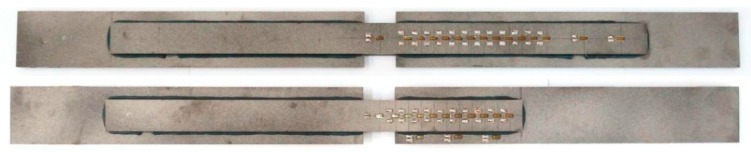
The specimens prepared for tests.

**Figure 2:**
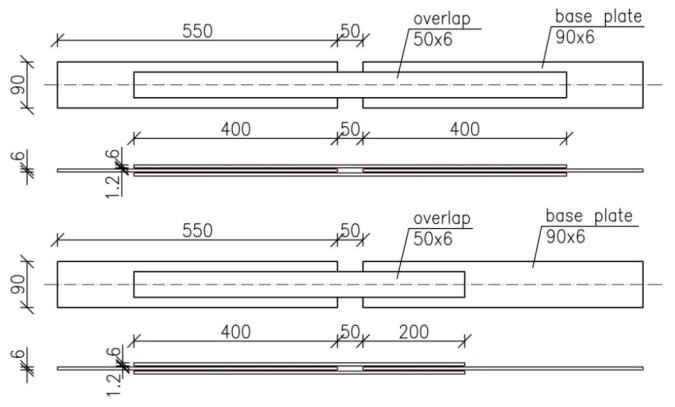
Detailed dimensions of the elements with an anchorage length of 400 mm and 200 mm.

Figure 1 presents the view of the specimens and Figure 2 shows the detailed dimensions of the elements with an anchorage length of 400 mm and 200 mm (bottom specimen).

### 2.2. Material Properties

The steel specimens were tested according to the EN ISO 6892:1-2016 recommendation [24]. The primary mechanical properties of the steel are summarized in Table 2.

The basic parameters of the methacrylate adhesive were adopted from the technical data sheet [25] and its details were taken from laboratory tests conducted by a parallel research group and presented in [26]. The most important parameters of the adhesive are summarized in Table 3. Other important material parameters were not considered in this study, but are already tested according to the recommendation given in the literature [27].

A great difference between the results provided by the manufacturer of the adhesive and the results of the studies was clear. This concerns, in particular, were the strain parameters of the adhesive, i.e., Young’s modulus and limit elongation at failure. These differences probably stem from the methodology of testing (various standards and type of test samples), however, such large quantities, mainly regarding the deformability of the adhesive, had to be fully verified—which has been done by a parallel research group.

Additionally, a standard pull-off test [28] was conducted in order to determine the bond strength of the MA adhesive to raw steel. The pull-off test on an aluminum disc was performed using a universal testing machine type MTS 810 (MTS Systems Corporation, Eden Prairie, MN, USA) because the range of a typical Dyna tester was insufficient to cause model failure. The pull-off force for a 50 mm diameter disc in three consecutive tests was 27.1, 30.1, and 25.8 kN, which corresponded to the bond strength of 13.8, 15.3 and 13.1 MPa, respectively. All the samples demonstrated an identical (adhesive) mode of failure; 60% of the failure surface involved separation of the adhesive from the steel surface, and 40% from the test disc.

### 2.3. Loading and Measurement System

In static series, the elements were subjected to axial tension applied by the universal testing machine type MTS 810 (MTS System Corporation, Eden Prairie, MN, USA) with the maximum force of 1000 kN. All specimens were loaded in one cycle, up to a failure, with a steady increase in the displacement of 1.27 mm/min (0.05 inch/min). The loading mode was adopted from the ASTM D3528-96 recommendation [24]. 

The traditional measuring equipment was used in all specimens. The following values were measured:

1. Tensile force at the yielding point and its maximum value; the yielding point was estimated based on the behaviour of the whole speciemen;
2. Total elongation of the specimen during loading. Note that specimen elongation included deformation of all steel elements and the adhesive; and
3. Strain at selected points located on the overlap and base plate surfaces.

**Figure 3:**
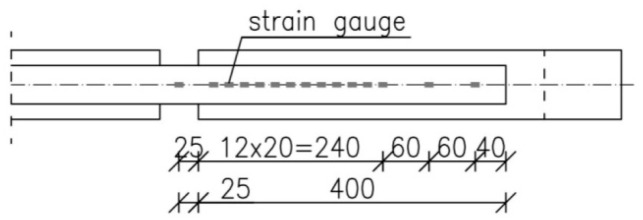
The arrangement of the strain gauges on the anchorage length of 400 mm.

Strain measurement was performed using foil strain gauges type 1-LY-11-6/120A (HBM, Darmstadt, Germany). The strain gauges were installed at intervals of 20 mm along the overlap (in specimens with 300 and 200 mm anchorage lengths). The location of the strain gauges in the specimens with the 400 mm anchorage length is shown in Figure 3, which shows the attenuation of the measuring points at the end of the overlap. 

In the specimens, S/DL/MA/RS-200-4/P2 and S/DL/MA/RS-400-4/P2, additional optical measurements were performed during the tests. They were performed on the whole surface of the specimen (on one side), so they involved one of the overlaps and both basic plates.

Optical measurements have been carried out using the CivEng Vision System developed by one of the authors at the Cracow University of Technology [29,30]. Digital images were processed by the original software and the results were obtained based on the digital image correlation method.

**Figure 4:**
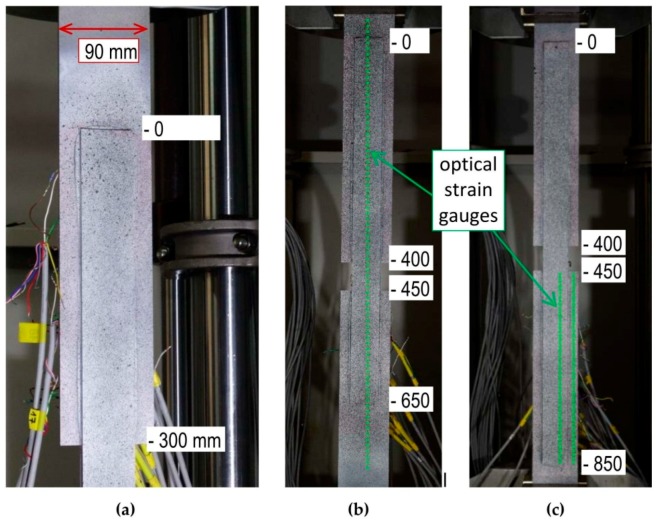
(**a**) Specimen prepared (painted) for optic measurement, (**b**) virtual measurement base used to calculate strain, and (**c**) base used to calculate displacement.

The DIC (digital image correlation) method is based on the correlation of the digital images obtained during the test, which are treated as a two-dimensional matrix consisting of pixels. Each pair of the pictures taken, respectively, before and after partial deformation are correlated and the points of the grid based on the specified image subsets (Figure 4) are matched and identified as that associated to the highest value of the correlation coefficient, which is calculated between the reference subset, *f*, and the target subset, *g*, whose dimensions are equal and are M × N pixels using the zero mean normalized cross correlation method, as described by the formula below:

$$CC^{ZMN}=\frac{\sum_{i=1}^{M}\sum_{j=1}^{N}\left(\left(f\left(i,j\right)-\mu_{f}\right)\times \left(g\left(i,j\right)-\mu_{g}\right)\right)}{\sqrt{\sum_{i=1}^{M}\sum_{j=1}^{N}{\left(f\left(i.j\right)-\mu_{f}\right)}^{2}}\times \sqrt{\sum_{i=1}^{M}\sum_{j=1}^{N}{\left(g\left(i,j\right)-\mu_{g}\right)}^{2}}}$$

where *μ_f_* means the intensity (luminosity) of the reference subset and *μ_g_* means the intensity (luminosity) of the target subset. 

Displacements of the image subsets obtained using DIC were originally computed in pixels and then converted into millimeters. To achieve this, the transition factor was used, calculated earlier using calibration patterns. Strain field was calculated from displacements measured at points of interest (grid points visible in Figure 4a). First, the region described by the convex hull of points of interest was triangulated. Using bilinear functions, the interpolation of displacement was constructed on resulting mesh. To obtain the strain values, derivatives in both the x and y direction were computed, and the strain components were as following [du_x/dx, du_y/dy, (du_y/dx + du_x/dy)].

Figure 4b,c present the arrangement of the virtual points, served as optical strain gauges, according to which the strains were calculated. The distances between those points were 18 mm (Figure 4b) and 9 mm (Figure 4c), depending on the measurement accuracy adopted.

## 3. Behaviour of the Specimen and Significant Values

**Figure 5:**
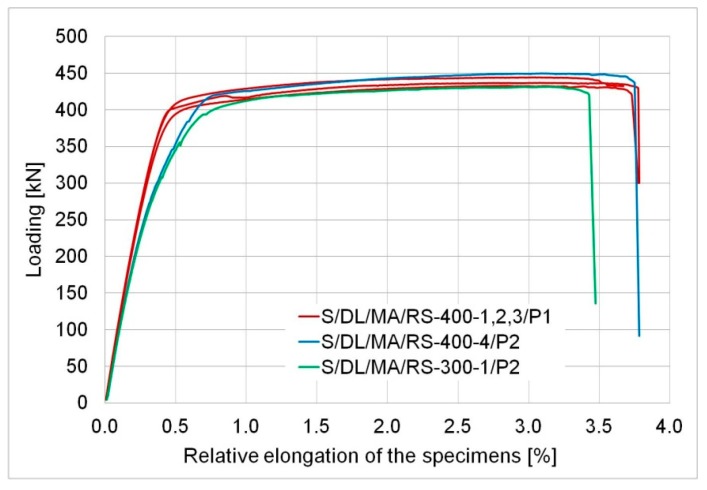
Relationship force—relative elongation of the specimens with the anchor length of 400 mm and 300 mm.

Two types of specimen behaviour were noted depending on the anchorage length. The first one, with a distinctive yield phase, where displacement increased rapidly at an almost-constant force, was noted for specimens with the anchorage length of 300 mm and 400 mm. This phenomenon is presented in Figure 5 as the relationship between the measured force and relative sample elongation calculated as a product of the elongation of the whole specimen and the initial grip spacing, which was 930 mm in all tested specimens. The sharp drops in the graphs (in the last phase) result from the specifics of the testing machine. Immediately after the failure of the specimen, the machine was switched off, but as previously noted, there was a small increase in jaw spacing with a sudden decrease in the force. 

For specimens of the S/DL/MA/RS-400 series, the following values were considered significant: The force at which the sample yielded with relevant stress values in the narrowest point of the sample (the lowest cross-sectional area of the basic plate) and the maximum tensile force with relevant stress values in the basic plate recorded during the test. The values are presented in Table 4. The values of the tensile stress (determined) measured upon the yield of the whole specimen was compared to the actual proportionality limit for steel (determined in material tests, see Table 1). As a consequence, plastic deformations occurred in the steel elements, which was confirmed by average permanent elongation values of the steel elements measured after the tests reaching 27 to 35 mm. Note that the steel used exhibited linear-elastic behaviour up to the stress of 750 MPa, which corresponds to a 405 kN force. At this point, a clear increase in sample length was recorded. The behavior of the components (yielding of the steel) indicates a “metal failure” (according to ASTM [23]), however, due to the specimen geometry adopted, the flat bar was not broken. Yielding of the basic plate led to a rapid increase in deformations of the adhesive layer, and resulted in its failure, which was a secondary effect. The initiation of the adhesive failure has been forced here, because the yielded section of the basic plate covered a part of the adhesive layer. With a high probability, it can be assumed that if the yielded section of steel were outside the adhesive joint (i.e., the length of the basic plate between the jaws of the testing machine and the edge of the overlap was significant longer—as recommended by the standard [23]), there would be no failure of the adhesive.

The elongation of the whole specimen was caused both by the yield in the steel elements and deformation in the adhesive layer that facilitated displacement of the overlaps in relation to the basic plates. The excessive deformation of the steel elements is undesirable for the user, because of the nature of their application (construction of the precise machines or structures). Therefore, the joint bearing capacity was considered the yield force (F_y_,_400_) of the specimen.

A particular variability of adhesive parameters related probably to its age was noted here. The second tested model (S/DL/MA/RS-400-4/P2) demonstrated a slightly greater deformability than the models bonded in the first series. This is indicated by the sample elongation upon yield (Table 4) and greater slope of the *F-ε* plot for model S/DL/MA/RS-400/P2. This fact does not, however, affect the recorded characteristic values (the yield and maximum tensile force) or the total elongation of the sample at failure (Table 4). In the case of the first series, the adhesive was applied right after purchase. Models in the second series were made using the same portion of the adhesive, which was stored for about one year at room temperature, which could affect its parameters.

A slightly lower yield force, maximum tensile force, and a smaller failure elongation of the specimen were measured for model S/DL/MA/RS-300 with the anchorage length of 300 mm than in specimens with the anchorage length of 400 mm. The load-bearing capacity of the element (which is its yield force) was only 3% lower than the average load-bearing capacity of models, where the anchorage length was 400 mm.

The behaviour of the elements in both cases was correct. It demonstrated that the load-bearing capacity of the adhesive was higher than the load-bearing capacity (here, it was identical to the force necessary to cause yield in the steel cross-section) of the weaker of the two bonded elements.

**Figure 6:**
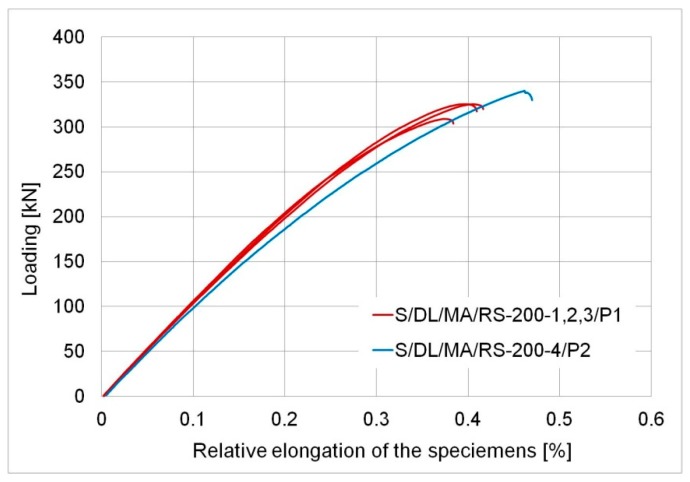
Relationship force—relative elongation of the specimens with the anchor length of 200 mm.

The other type of behaviour of the specimens was noted for the anchorage length of 200 mm. All the samples exhibited elastic behaviour until failure, which is shown in Figure 6, presenting the relationship between the force and average elongation of the specimen (calculated in relation to the initial grip spacing). No plastic deformation of the steel elements or the adhesive was recorded. The failure was sudden and violent, in the form of adhesive failure, i.e., debonding of the adhesive from the steel surface. The following values were considered reliable values for specimens, S/DL/MA/RS-200: The maximum tensile force (and relevant tensile stress values for the basic plate), considered the joint load-bearing capacity in the study, and the ultimate elongation for this force. The values are presented in Table 5. It is clear that the 200 mm anchorage length is insufficient to ensure the full load-bearing capacity of the joint resulting from exceeding the capacity of the bonded elements, which here resulted from the yield of the steel. The load-bearing capacity of elements with 200 mm overlap anchorage was about 20% lower than for models with a 400 mm length. Thus, the described nature of failure directly corresponds to the “adhesive failure”, according to ref. [23].

It should be noted here that the investigated samples were made of high-strength steel. In the case of plates made of typical structural steel, S235 or S355, a 200 mm anchorage would be enough to cause yield in the basic plate if geometric parameters were maintained (the estimated yield force was 195 kN and 275 kN, respectively).

This group of models also demonstrated a slight variability of parameters of the adhesive in the second series specimen (signed as P2), involving a slight increase in its deformability. It is clearly visible on the force-relative elongation graph (Figure 6) and the measured elongation at the break of sample S/DL/MA/RS-200-4/P2.

## 4. Mode of Failure

Two issues related to the failure of the elements are discussed. The first one concerns the final failure pattern of the specimens. The second one describes a detailed mechanism of the failure (nature of failure in terms of the standard [23]), which depends on the anchorage length used.

**Figure 7:**
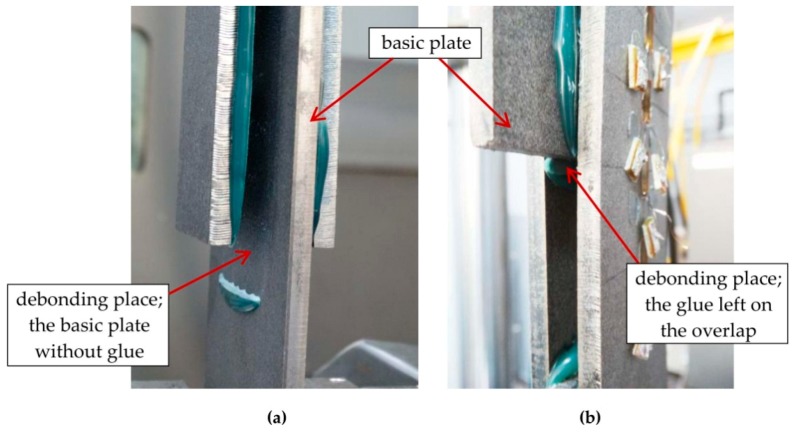
Places of the debonding: (**a**) At the end of the overlap (specimens with the anchorage length of 400 mm), (**b**) in the middle of the specimen (specimens with the anchorage length of 200 mm).

**Figure 8:**
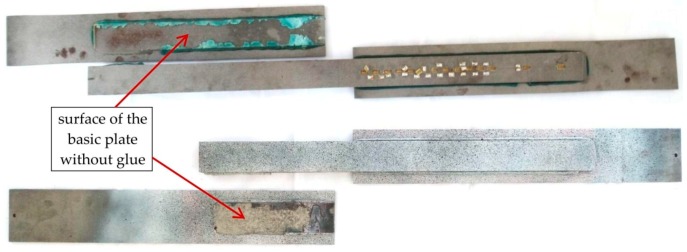
The specimens after failure: Above the element from series S/DL/MA/RS-400/P1 and below the element from series S/DL/MA/RS-200-4/P2.

In all groups of elements, the final failure pattern was very similar—the connection between the basic steel plate and the adhesive layer was broken. The places of debonding of the adhesive from the flat bar are visible in Figure 7a,b. In elements with the anchor of 400 mm, the debonding was initiated at the end of the overlap where the basic element yielded (Figure 7a). In specimens with the anchorage length of 200 mm, the debonding of the adhesives was initiated in the middle of the model where the basic element ended (Figure 7b). The debonding of the adhesive was noted in all the cases between the surface of the steel and the adhesive. The dominant failure surface was the interface between the adhesive and the substrate element as 70% to 90% of the adhesive remained on the overlaps. This was the case for all types of elements. The images of the adhesive layer surface left on the substrate flat after the failure is shown in Figure 8 where picture (a) shows an S/DL/MA/RS-400/P1-group sample and picture (b), sample S/DL/MA/RS-200-4/P2. The difference in adhesive color results from the specimens’ storage conditions after the test.

In contrast to the final failure pattern, two different natures of failure identified in the specimens clearly differentiated by the fact of reaching the yielding point of steel, or not, of the basic plate. On the basis of the standard [23] in the specimens in which the yielding of the steel occurred (specimens with 300 and 400 mm anchorage lengths), the “metal failure” was determined. The failure of the adhesive observed after the yielding of the steel was a secondary here, which results directly from the geometry of the specimen. In the sample recommended in [23], the section of the basic plate between the jaws of the testing machine and the edge of the overlap had a length of 63.5 mm and a width of 25.4mm, hence the ratio was 2.5:1. In the specimen tested, this proportion was 0.55:1, which makes it impossible to completely plasticize the flat bar on its free section, outside the overlap. In specimens with a 200 mm anchoring length, the debonding between the adhesive and steel plate occurred, which qualifies the nature of failure as the “adhesive failure”.

**Figure 9:**
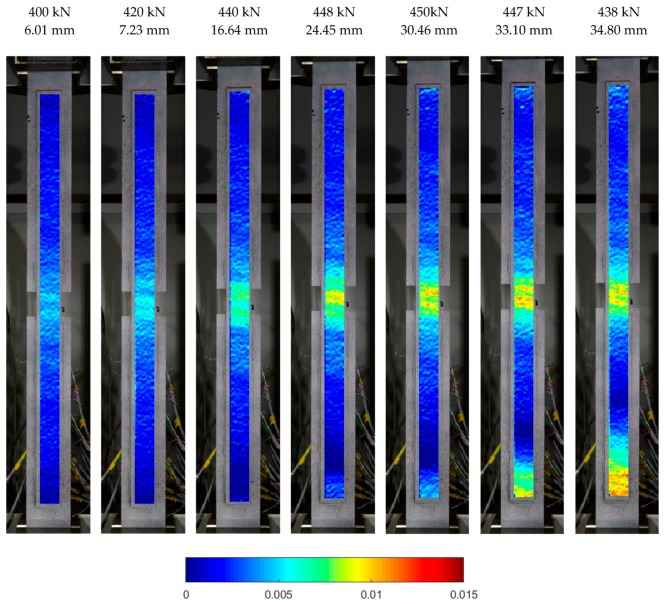
Longitudinal strain distribution maps at yielding and failure in S/DL/MA/RS-400-4/P2.

**Figure 10:**
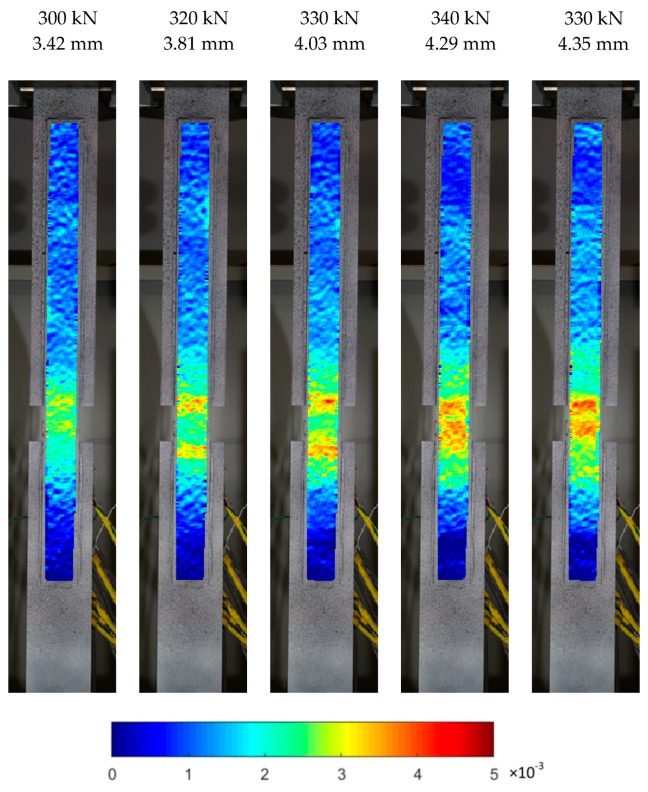
Longitudinal strain distribution maps at failure in S/DL/MA/RS-200-4/P2 specimen.

This results in a different way of reaching the limit parameters of the glue, exceeding that which caused the failure of the joint. The failure mechanism for models, S/DL/MA/RS-400-4/P2 and S/DL/MA/RS-200-4/P2, is clearly visible on longitudinal strain maps developed thanks to optical measurements. Relevant strain maps are shown in Figure 9 and Figure 10. The maps are shown for selected loads (taken from the testing machine) and the corresponding total elongation of the specimen measured in the testing machine. The color changing (from blue to the red) means a gradual increase in the local longitudinal strain.

Figure 9 shows the longitudinal strain distribution in the steel overlap at the anchorage length of 400 mm during the yield phase and at the ultimate failure. The parts of images with the basic plates were blanked to improve clarity. It is clearly visible that up to the force of 440 kN, the strain was increasing in the central zone of the element to reach around 0.7%. Above this force value, which is virtually in the failure phase, the strain in the lower end of the overlap started to grow, which was described in point 3 as an effect of the yield in the basic plate in this zone. After the maximum force was reached, the whole specimen yielded (cf.: Graph in Figure 5) and the end of the overlap reached high strain values (ca., 1.2 %) comparable to those in the central zone. This demonstrated that the adhesive joint retained its full load-bearing capacity even in this phase as it was able to transfer such large strains from the basic plate to the end of the overlap.

Detailed recognition of the failure mode of the specimens was possible only through the use of optical measurements. The tensometric measurement, due to the limited number of sensors and their localization, was not able to record it. 

Figure 10 presents a specimen with the anchorage length of 200 mm. Successive longitudinal deformation maps show a failure initiated in the central zone of the element (compared with Figure 7b). This is compatible with the behaviour of specimens with 400 mm of anchorage length before the yield of the basic plate, which did not occur in samples with the anchorage length of 200 mm. The strain reached at failure amounted to ca. 0.5%, which is equal to the strain value recorded in specimens with an anchorage length of 400 mm (Figure 9) just before the yield phase (force value of 400 kN).

**Figure 11:**
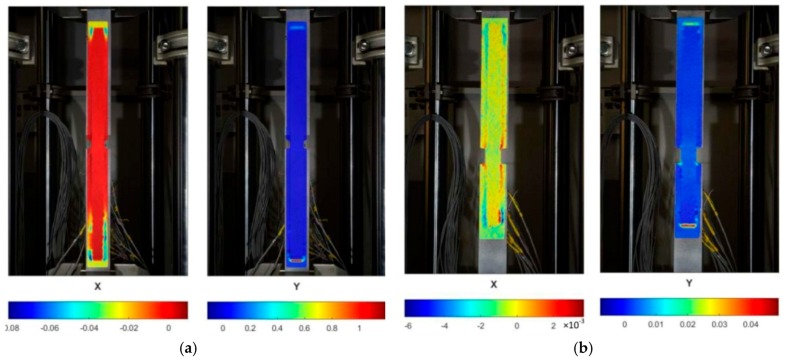
Strain distribution maps at final failure (horizontal direction—X, vertical direction—Y): (**a**) Specimen S/DL/MA/RS-400-4/P2—438kN, (**b**) specimen S/DL/MA/RS-200-4/P2—330kN.

Figure 11 shows coherent strain maps of overlaps and basic plates at failure. The X-direction means strain transversal to the force (horizontal in the figure) and the Y direction is the strain consistent with the force vector direction (along the element). In the case of the specimen with the anchorage length of 400 mm, a strong necking of the basic plate is clearly visible near the grips of the testing machine, indicating a yield of this element. In the 200 mm-anchorage specimen, the values are one order lower, which means the steel exhibited elastic behaviour. In both cases, there are visible very strong strains in adhesive bands at the front of the overlaps, which locally exceed 100% in the very loaded sample, S/DL/MA/RS-400-4/P2. The above-mentioned observations are consistent with the sample failure scheme described above.

Generally, the presented maps indicate that in a given material (high-strength steel) and geometric condition, the anchorage length of 200 mm is insufficient to create an effective joint (the adhesive layer failed), and 400 mm provides the capacity of adhesive layer greater that the capacity of jointed elements. From a single test of a specimen with a 300 mm anchorage length, it could be noted that under the tested conditions, this bonding length seems to be long enough to make a full load-bearing joint.

## 5. Development of the Shear Strains

### 5.1. Comparison of the Tensometrical and Optical Measurements

A significant part of the conclusions for the specimen failure mechanism was based on optical measurements of the strain. As was mentioned before, an original method for optical measurements was applied here [29,30] as described in Section 2.3. The measurements were made for three specimens of the P2 series. The probing frequency, limited by the capability to take and record very high-resolution images, was, in a way, a drawback of the system. It was not an issue in this particular case as the displacement increment was very slow (1.27 mm/min) and a single image was taken at about every 0.02 mm of displacement of the universal testing machine head.

A classic electrical resistance strain gauge method (tensometric method) was used to measure the strain of every specimen (for the description of strain gauges locations, see Section 2.3). Despite all its advantages (including a high reliability of results), this measurement system suffered from a significant disadvantage, which was the limitation of the results caused by a limited number of strain gauges per model (which, in turn, resulted from the limitations of instruments).

**Figure 12:**
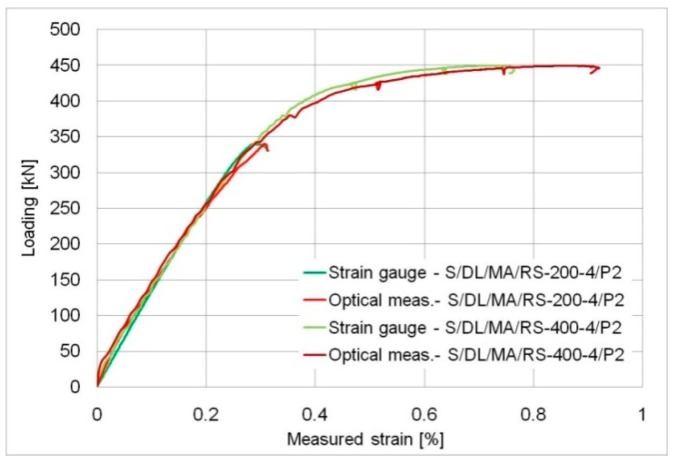
Comparison between the values measured using strain gauges and optical method.

As a result of the application of this measuring method, it was possible, on the one hand, to determine the repeatability of the results (based on an analysis of strain measurements) and, on the other hand, to compare results from both types of measurements, thus, in a way, verifying the original optical method. The comparison was performed for the center of the investigated element, i.e., at the locations where the basic plates broke, on overlaps. The results are shown in Figure 12.

The green plots show strain gauge results and red plots show the results of virtual optical strain gauges located near the centre of the element. The strain plots from the strain gauges and optical measurements were similar over almost the whole course of the experiment. It was not until the failure phase (clearly outside the range of the Hooke’s law) that the optical measurement indicated a slightly larger strain than the strain gauges. Note that the strain gauge and optical measurements were made on the opposite sides of the element (on different overlaps) and, therefore, their results may differ due to the unavoidable axial misalignment of the specimen, which was most apparent during the failure phase.

The accuracy of the optical measurement is comparable with traditional measurement systems, but optical measurement facilitates the generation of strain-stress curves for more points simultaneously without using any additional physical sensors.

### 5.2. Distribiutions of Strains and Displacements (Slip)

Due to the comprehensive nature of the optical measurements involving the whole investigated element, further strains resulted from this measuring system. The above-mentioned conformity with the results of typical strain gauge measurements fully substantiates this approach.

**Figure 13:**
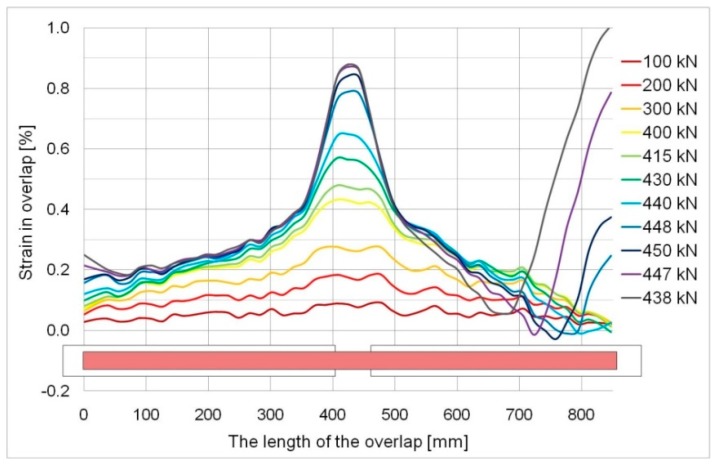
The strains determined over the whole overlap length, specimen S/DL/MA/RS-400-4/P2.

**Figure 14:**
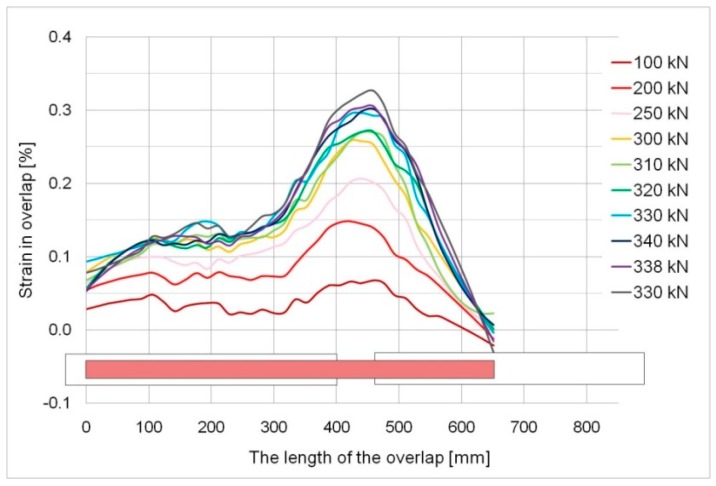
Strains determined over the whole overlap length, specimen S/DL/MA/RS-200-4/P2.

Figure 13 and Figure 14 show strain graphs over the whole overlap length for specimens, S/DL/MA/RS-400-4/P2 and S/DL/MA/RS-200-4/P2. Small areas of the negative strain values can be seen in the diagrams. In the case of Figure 14, in the initial loading phase, these values result from small initial imperfections of the elements. In both graphs (Figure 13 and Figure 14), such values are also visible in the failure phase—this is due to uneven failure of the adhesive layer on both sides of the specimen, and thus from the appearance of the temporary eccentricity of the force, which causes local bending. This is evidenced by the fact of the shift of the negative values zone visible in Figure 13, along with the progressive yielding of the overlap (just prior to the failure of the adhesive).

**Figure 15:**
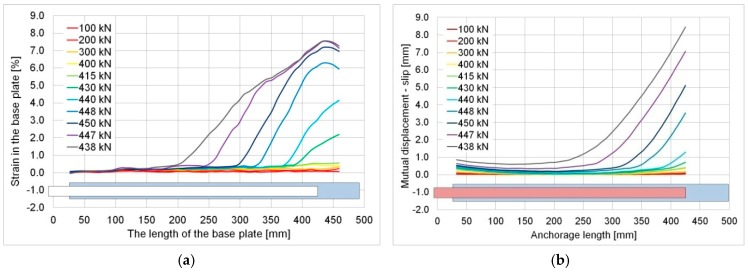
Specimen S/DL/MA/RS-400-4/P2: (**a**) Strain measured in the basic plate, (**b**) calculated slip between the overlap and basic plate.

In both cases, there is a rather typical strain distribution observed for smaller loads. The strain reached the maximum value in the middle of the specimen, where the continuity of the basic plate was broken, i.e., where the overlaps do not work with the base. Next, the strain gradually decreased over the length of the overlaps to reach the lowest value near where the overlap ended. In the case of the element with the effective anchorage length (400 mm), this changed rapidly directly before the failure phase when a rapid increase in strain took place near an end of the overlap (cf. Figure 9). This seems illogical, but can be easily explained by the yield of the basic element. A rapid increase in the strain in the base caused the observed increase in the strain in the ends of the overlap. This confirmed a huge ability of the adhesive to transfer high deformation of the basic steel element into the overlap. The final failure occurred when the strain exceeded the shear deformability (strength) of the 1.2 mm thick layer of adhesive. It is clearly visible in Figure 15a, which shows the behavior of the basic plate at selected load levels. As was mentioned above, the adhesive failed as a result of the enforced significant deformations, which was shown in the slip values calculated as a mutual dislocation between the overlap and basic plate—Figure 15b. Within the interval of the elastic steel strain, up to 400 kN of load, the mutual displacements over the length of the joint did not exceed 0.5 mm and were the greatest in the central part of the element. They grew rapidly at larger loads, in particular near the end of the overlap. All this builds a coherent image of a failure for the 400 mm anchorage length specimen in which the adhesive ensures full load-bearing capacity of the joint over the whole interval of elastic behaviour of steel elements (at low strains). Then, together with the yielding of the basic steel, the adhesive layer exhibited a plastic behaviour, which allowed the transfer of significant deformation (Figure 15b). Finally, the further increase in deformation led to the sudden failure of the adhesive, in which the ultimate shear strain was exceeded. No progressive failure of the adhesive was observed. The described behavior is clearly visible on the relevant strain maps (cf. Figure 9).

**Figure 16:**
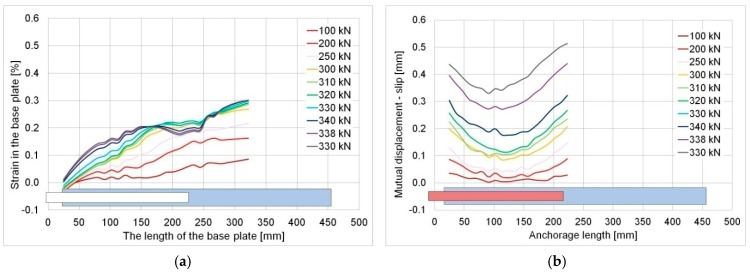
Specimen S/DL/MA/RS-200-4/P2: (**a**) Strain measured in the basic plate, (**b**) calculated slip between the overlap and basic plate.

In the case of the 200 mm overlap, the failure took place in the elastic phase at the steel so the above-mentioned change in the strain system did not happen. Thus, the direct cause of the failure was the fact that the load-bearing capacity of the adhesive joint was exceeded, which indicates that its length was insufficient (for the specific geometry and steel grade). In this case, a quasi-brittle failure occurred, which is clear from the violent course at a relatively low (below 0.5 mm) displacement of overlaps in relation to the basic plate (Figure 16a,b). It was confirmed in the optical measurement results (cf. Figure 10).

Note that the strain values in both elements were comparable at the same load level.

## 6. Conclusions

Adhesive joints are not very popular in the construction industry today. They usually are second to other fastening methods or are used to strengthen the structure. A typical solution is to glue FRP laminates to steel structural components. This approach makes use of epoxy adhesives dedicated to specific joint systems. The drawback is high stiffness, which causes stress concentration at the ends of the joined elements and brittle failure of the joint. It is, however, justified insofar as the laminate matrix is also an epoxy resin, the parameters of which are comparable to the adhesive.

In steel-to-steel joints, the epoxy resin is too stiff and brittle, which does not ensure full use of the capacity of the joined elements. In such a case, the selection of the appropriate adhesive becomes the key issue. This problem was previously considered, first on the basis of a literature analysis and then in preliminary comparative tests [19] that included a group of methacrylate adhesives, available on the Polish market. As a result, the one with the best projected features was presented in this study.

The present study experimentally confirmed the appropriateness of the use of a methacrylate adhesive in steel-to-steel joints made of high-strength steel and subjected to static loads. The tests were carried out on elements with dimensions comparable to actual construction elements to minimalize size-effect influences. This is important because a number of tests available in the literature were performed on very small samples, which may cause overestimation of the capacity.

The main conclusions derived from the analysis are:

1. The bonding length, ranging between 300 mm and 400 mm, and the adhesive layer thickness of 1.2 mm provide the capacity of the adhesive joint to be higher than the capacity of a single steel component;
2. The specimen behavior depended on the anchorage lengths and resulted in quasi-brittle behavior of the models in the case of 200 mm anchorage, and ductile behaviour, when the anchorage length exceeded 300 mm;
3. The nature of failure, according to the standard [23], is strongly related to the anchorage length; in specimens with a 200 mm anchorage length, the adhesive failure was observed, while in specimens with 300 and 400 mm anchorage lengths, the metal failure (steel yielding) occurred and the failure of the adhesive (debonding) was a secondary effect;
4. The longitudinal deformability of the adhesive was relatively high, as evidenced by the possibility of transferring the deformation from the yielded base steel plate to the overlap.

At present, further laboratory tests are being carried out to determine the behavior of identical steel-to-steel joints (made with the same methacrylate adhesive) subjected to cyclic loads in fatigue tests. The results will be presented in the next paper. At the same time, the parallel research group is carrying out in-depth material tests of the methacrylate adhesive used (according to different standards), including analyses to help predict the aging of the adhesive in a joint. The obtained data will be used to create a numerical model. It will be validated using the results of the research presented in this paper and the results of fatigue tests. This effort will result in a numerical and analytical model to predict the behavior of various types of structural joints where the tested adhesive is used under various loads and exploitation conditions.

## Figures and Tables

**Table 1:** Designation of the specimens.

Specimen	Number of Specimens Tested	Anchorage Length (mm)	Part of the Adhesive
S/DL/MA/RS-200-i/P1	3	200	P1
S/DL/MA/RS-200-i/P2	1	200	P2
S/DL/MA/RS-300-i/P2	1	300	P2
S/DL/MA/RS-400-i/P1	3	400	P1
S/DL/MA/RS-400-i/P2	1	400	P2

**Table 2:** Main steel properties.

Proportional Limit Stress [MPa]	Yield Strength (MPa)	Tensile Strength (MPa)	Ultimate Elongation (%)
~750	791.7	825.8	21.5

**Table 3:** The most important parameters of methacrylate adhesive (MA).

	Speed of Loading (mm/min)	Tensile Strength (MPa)	Shear Strength (MPa)	Modulus of Elasticity (MPa)	Ultimate Elongation (%)	Poission’s Ratio
Producer data	unknown	18.6–20.7	>14	517–689	30–50	0.41
Data taken from [26]	1.0	14.70	-	1058	8.35	0.37
	10.0	16.21	-	1131	6.81	-

**Table 4:** Static test results for the specimen with 400 mm and 300 mm anchorage.

Specimen	Yield Force F_y,400; y300_ (kN)	Yield Stress σ_y,400; y300_ (MPa)	Elongation at Yielding, ε_y400; y300_ (%)	Max. Force F_u,400; u,300_ (kN)	Max. Stress σ_u,400; u,300_ (Mpa)	Elongation at Failure, ε_y400; y300_ (%)
S/DL/MA/RS-400-1/P1	402	744	0.50	436	807	3.78
S/DL/MA/RS-400-2/P1	398	737	0.53	432	800	3.73
S/DL/MA/RS-400-3/P1	409	757	0.52	443	820	3.67
S/DL/MA/RS-400-4/P2	418	774	0.76	450	831	3.75
*Average value:*	*406*	*752*	*-*	*440*	*815*	*3.73*
S/DL/MA/RS-300-1/P2	393	728	0.69	431	798	3.11

**Table 5:** Static test results for the specimen with 200 mm anchorage.

Specimen	Max. Force F_u,200_ (kN)	Max. Stress σ_u,200_ (MPa)	Elongation at Max. Force, u_u,200_ (mm)
S/DL/MA/RS-200-1/P1	308	570	3.5
S/DL/MA/RS-200-2/P1	325	602	3.8
S/DL/MA/RS-200-3/P1	325	602	3.7
S/DL/MA/RS-200-4/P2	340	630	4.3
*Average value:*	*325*	*601*	*-*

## References

[1] Ladinek M., Niederwanger A., Lang R., Schmid J., Timmers R., Lener G. (2018). The strain-life approach applied to welded joints: Considering the real weld geometry. J. Constr. Steel Res..

[2] Fallahnezhad K., Steele A., Oskouei R. (2015). Failure Mode Analysis of Aluminium Alloy 2024-T3 in Double-Lap Bolted Joints with Single and Double Fasteners; A Numerical and Experimental Study. Materials.

[3] Chen N., Luo H., Wan M., Chenot J. (2014). Experimental and numerical studies on failure modes of riveted joints under tensile load. J. Mater. Process. Technol..

[4] Zhao X., Wang M., Zhang Z., Liu Y. (2016). The Effect of Ultrasonic Peening Treatment on Fatigue Performance of Welded Joints. Materials.

[5] Guo H., Wan J., Liu Y., Hao J. (2018). Experimental study on fatigue performance of high strength steel welded joints. Thin-Walled Struct..

[6] Pasternak H., Schwarzlos A., Schimmack N. (2004). The application of adhesives to connect steel members. J. Constr. Steel Res..

[7] Kaya A., Tekelioglu M. (1998). Three Dimensional Stress Analysis in adhesively Bonded Joints. Math. Comput. Appl..

[8] Wong E.-H., Liu J. (2017). Design Analysis of Adhesively Bonded Structures. Polymers.

[9] Da Silva L.F.M., das Neves P.J.C., Adams R.D., Spelt J.K. (2009). Analytical models of adhesively bonded joints—Part I: Literature survey. Int. J. Adhes. Adhes..

[10] De Morais A.B., Pereira A.B., Teixeira J.P., Cavaleiro N.C. (2007). Strength of epoxy adhesive-bonded stainless-steel joints. Int. J. Adhes. Adhes..

[11] Fawzia S., Zhao X.-L., Al-Mahaidi R. (2010). Bond–slip models for double strap joints strengthened by CFRP. Compos. Struct..

[12] Dehghani E., Daneshjoo F., Aghakouchak A.A., Khaji N. (2012). A new bond-slip model for adhesive in CFRP–steel composite systems. Eng. Struct..

[13] Majidi H., Razavi S., Berto F. (2017). Failure Assessment of Steel/CFRP Double Strap Joints. Metals.

[14] Reis P.N.B., Ferreira J.A.M., Antunes F. (2011). Effect of adherend’s rigidity on the shear strength of single lap adhesive joints. Int. J. Adhes. Adhes..

[15] Markolefas S.I., Papathanassiou T.K. (2009). Stress redistributions in adhesively bonded double-lap joints, with elastic–perfectly plastic adhesive behavior, subjected to axial lap-shear cyclic loading. Int. J. Adhes. Adhes..

[16] Akisanya A.R., Fleck N.A. (1992). Brittle fracture of adhesive joints. Int. J. Fract..

[17] Moreira D.C., Nunes L.C. (2014). Experimental analysis of bonded single lap joint with flexible adhesive. Appl. Adhes. Sci..

[18] Rajkumar G.R., Krishna M., Narasimhamurthy H.N., Keshavamurthy Y.C. (2017). Statistical Investigation of the Effect of Process Parameters on the Shear Strength of Metal Adhesive Joints. J. Inst. Eng. Ser. C.

[19] Hulimka J., Kałuża M. (2017). Preliminary Tests of Steel-to-steel Adhesive Joints. Procedia Eng..

[20] Kałuża M., Hulimka J., Kubica J. (2015). Effectivness of adhesive CFRP/steel joints double-lap static tests. Proc. Int. Symp. Brittle Matrix Compos..

[21] Lopez-Cruz P., Laliberté J., Lessard L. (2017). Investigation of bolted/bonded composite joint behaviour using design of experiments. Compos. Struct..

[22] Arnautov A., Nasibullins A., Gribniak V., Blumbergs I., Hauka M. (2015). Experimental Characterization of the Properties of Double-Lap Needled and Hybrid Joints of Carbon/Epoxy Composites. Materials.

[23] (2016). ASTM D3528-96: Standard Test Method for Strength Properties of Double Lap Shear Adhesive Joints by Tension Loading.

[24] (2016). ISO 6892-1:2016: Metallic Materials: Tensile Testing—Part 1: Method of Test at Room Temperature.

[25] PLEXUS—Technical Data Sheet. https://www.techsil.co.uk/media/pdf/TDS/ITEP14042-tds.pdf.

[26] Kozłowski M., Bula A., Hulimka J. (2018). Determination of mechanical properties of methacrylate adhesive (MMA). ACEE.

[27] Da Silva L.F.M., Dillard D.A., Blackman B.R.K., Adams R.D. (2012). Testing Adhesive Joints: Best Practices.

[28] (2017). ASTM D4541-17: Standard Test Method for Pull-Off Strength of Coatings Using Portable Adhesion Testers.

[29] Tekieli M., Słoński M. (2013). Application of Monte Carlo filter for computer vision-based bayesian updating of finite element model. Mech. Control.

[30] Tekieli M., Słoński M. (2015). Digital image correlation and Bayesian filtering in inverse analysis of structures. Recent Adv. Civ. Eng. Comput. Methods.

